# Gut eutrophication

**DOI:** 10.3389/frmbi.2024.1481250

**Published:** 2024-12-04

**Authors:** Chika Edward Uzoigwe

**Affiliations:** Department of Science, Harcourt House, Sheffield, United Kingdom

**Keywords:** gut eutrophication, gut microbiome, metabolic disease, obesity, diet, sugar, exercise

## Abstract

“Classical eutrophication” occurs when raw unfixed nutrients enter an aquatic environment. This causes the deleterious proliferation in fauna most adept at exploiting this abundance of nutrition. The net effect is de-diversification. We propose an analogous process in the gut: “gut eutrophication”. Evidence shows that consumption of processed food, high in unfixed disaccharides, causes an expansion of bacteria in the gut habitat with a metabolic proclivity for these nutrients. This is at the expense of microbiota with a predilection for complex macromolecule macronutrients. There is a loss of diversity and the effect is exacerbated by a sedentary lifestyle. Gut luminal low oxygen tension favors salubrious gut commensals. This effect is potentiated by exercise but thwarted by inactivity. Antibiotics cause an obvious gut dysbiosis. So too can diet in a more insidious manner. The transition in microbial composition, seen in “gut eutrophication”, may be an aetiological component of metabolic disease-associated gut dysbiosis.

## Introduction

There exists the fundamental assumption that hosts act to optimise the microbial composition in order to promote physiological function and, in particular, do so behaviourally (Wilde et al., 2024). This is not necessarily the case. Indeed, at times hosts act to sabotage and undermine the henotic microbial symbiosis. This is typically seen in a human host and is the bedrock of many non-communicable diseases. The consumption of refined substrates elicits the proliferation of commensals and/or symbionts, most adept at metabolising such raw oligomer materials (Ross et al., 2024). These tend to be species with celeritous and fugacious lifecycles. In their rapid rise and overgrowth, they out-compete and subdue their peers, critically leading to microbial de-diversification (Ross et al., 2024). This is “gut eutrophication”. The progeny of this process is not those most conducive to host physiological eudemonia. Simply put, diet impacts the gut microbiome. The human diet is often dictated by palatability and appetite for processed foods rather than the exigencies of the gut habitat. Humans are most acquisitive of monosaccharides, disaccharides, and salt (sodium chloride). Such food substances, when found in nature are delivered in a healthy composite. Hence, during evolution, halo- and glyco-petal proclivity would have been salubrious to the gut habitat and physiological performance. Halotropic and glycotropic behaviours would have been copacetic phenotypes, largely due to indigence in these substrates. Today, however, two significant human innovations make these behaviours/traits injurious. The first is animal and plant husbandry resulting, for many, in the nimiety of such substrates. Secondly, and most significantly, is industrial food production. Sugar and salt are extricated from their healthy co-constituents. They are refined and concentrated. Thus, unlike other species, we have the ability to match this insatiable appetite for salt and di/mono-saccharides with a panoply of diverse high-sugar high-salt foodstuffs. There is discordance between human preferences based on palatability and substrates which actually foster a gut habitat most conducive to host health (Ross et al., 2024).

## Aquatic and gut eutrophication

**Figure 1:**
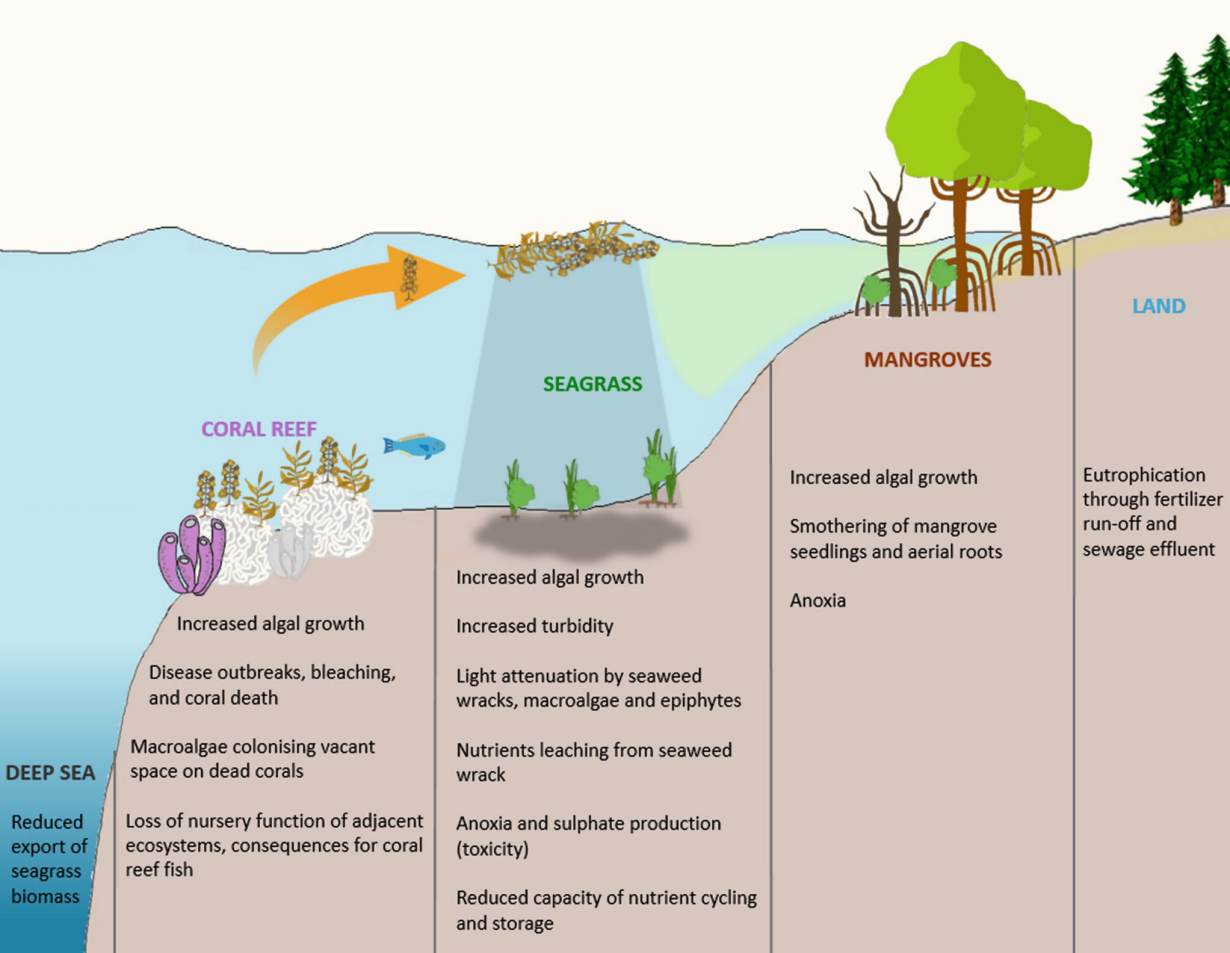
The process of aquatic eutrophication. Figure from: Consequences_of_eutrophication_on_coral_reef,_seagrass_and_mangrove_ecosystems.png (2044×1583).

In natural habitats, there exists finite resources and competition between species. Those that achieve durable existence are those that not only most efficaciously use the resources available but promote the very health of the habitat rather as opposed to those that pursue aggressive sequestration of resources and follow a Ponzi-style lifecycle. The unrestricted introduction of elemental resources can disrupt the ecological equilibrium and result in harm to the habitat. In an aquatic habitat, this is observed in the form of eutrophication. Classically, eutrophication occurs when fertilisers and micronutrient-rich effluent enters an aquatic milieu. This results in the proliferation of algae with celeritous lifecycles. This is injurious to the environment. The algae sequester oxygen, obfuscate light penetration, resulting in the death of plant fauna. The algae themselves ultimately expire. The degradation of these plant species further deprives the habitat of oxygen, rendering it uninhabitable (Figure from: Consequences_of_eutrophication_on_coral_reef,_seagrass_and_mangrove_ecosystems.png (2044×1583)) (**Figure 1**).

**Figure 2:**
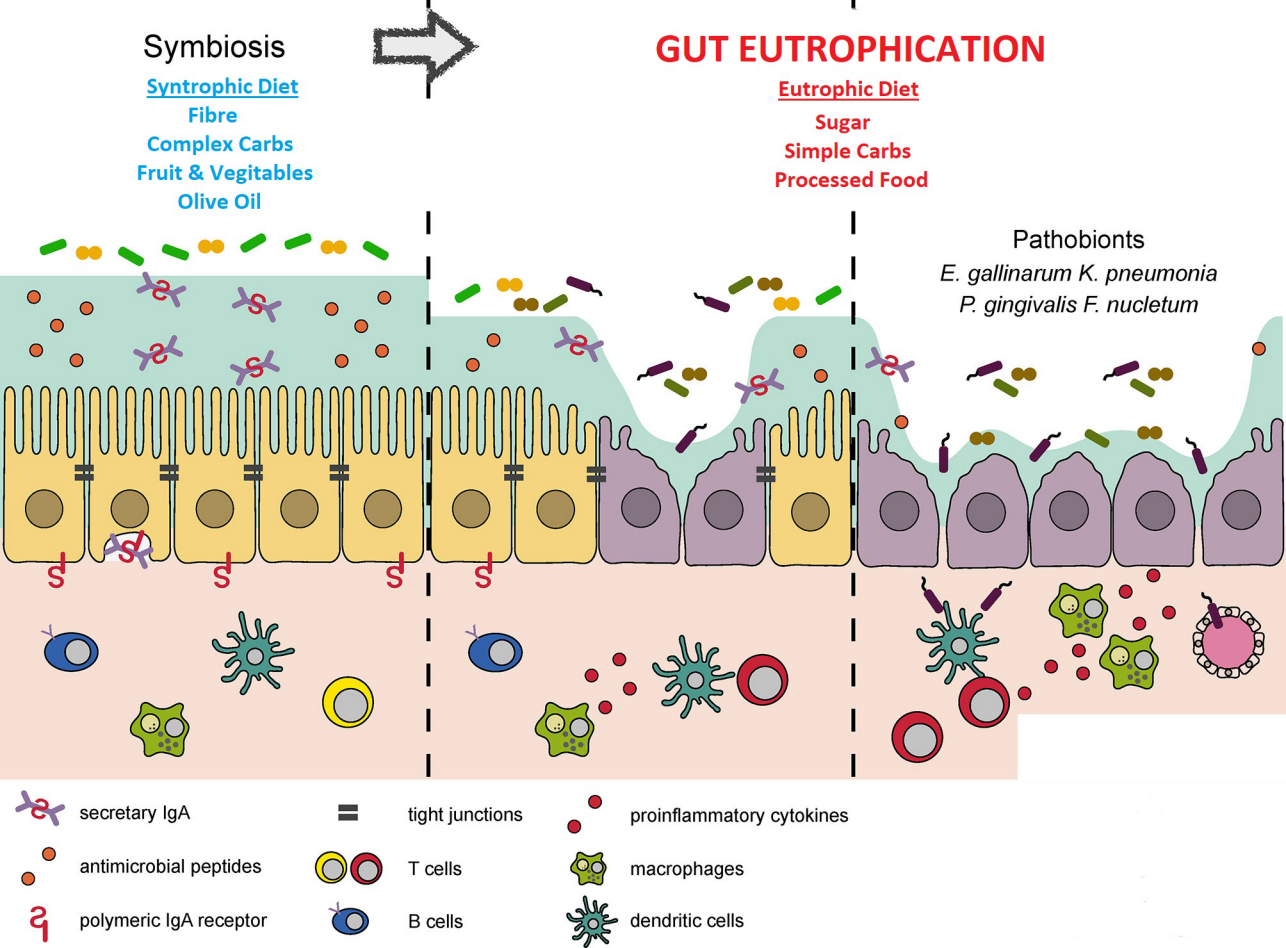
The process of gut eutrophication. Adapted from Kinashi Y, Hase K. Partners in Leaky Gut Syndrome: Intestinal Dysbiosis and Autoimmunity. Front Immunol. 2021 Apr 22;12:673708.

The human gut is a habitat. It is suggested that an analogous process can occur here, resulting in a human disease, notably metabolic disease. This is the gut eutrophication hypothesis. Exposure of the gut to high concentrations of raw unfixed nutrients results in “gut eutrophication”. Kawano et al. showed in murine models that a high-sugar diet resulted in overgrowth of *Faecalibaculum rodentium* including *Erysipelotrichaceae* bacteria (Kawano et al., 2022). This was at the expense of bacteria that exhibited favourable immuno-tropic characteristics that would promote euglycaemia and prevent metabolic syndrome (Kawano et al., 2022). A further consistent finding is that high sugar alimentation, analogous to eutrophication, results in a reduction in gut diversity (Zoetendal et al., 2012; Do et al., 2018). The pre-eminent species following gut eutrophication tend to be pro-inflammatory. The patho-mechanism is similar to that of aquatic eutrophication. Commensals that flourish during gut eutrophication are those adept at metabolising raw ingredients with celeritous lifecycles (Satokari, 2020; Takeuchi et al., 2023). These rise to ascendancy over gut bacteria specialised at degrading complex carbohydrates and have more protracted life cycles (**Figure 2**: Adapted from: Kinashi Y, Hase K. Partners in Leaky Gut Syndrome: Intestinal Dysbiosis and Autoimmunity. Front Immunol. 2021 Apr 22;12:673708).

Eutrophication derives its etymology from the Greek words “*ϵὖ*” and “*τροϕικός*” meaning “well-fed” or “well-nourished”. In the aquatic milieux, the nimiety of nutrients is frequently in the form of nitrates and phosphates from fertiliser. In the gut, the surplusage is typically in the guise of excess processed sugars such as monosaccharides and disaccharides. However, given that the gut lumen is anoxic, it may also be oxygen. This is of relevance in the consideration of the impact of exercise or lack thereof on gut fauna.

## Exercise and gut eutrophication

Beneficiaries of the eutrophic milieux move to maintain the dysglycemic environment. Recent work has shown that the gut microbial profile also affects the motivation of mice to engage in physical exercise (Agirman and Hsiao, 2022; Dohnalová et al., 2022). This proclivity for and performance during exercise can actually be transferred between mice by transplanting the gut microbial fauna from high-performance mice to mice bred with a sterile gut. The latter then adopt the motivation and performance of the donors (Agirman and Hsiao, 2022; Dohnalová et al., 2022). Microbial fauna can thus remarkably manipulate the environment to promote their own existence. This is directly analogous to the fauna that are predominant following aquatic eutrophication that produce a similar phenomenon by means of their oxygen sequestration and tenebrific effect.

A lack of physical activity, like a poor diet, is a second behavioural choice that profoundly impacts gut microbial phenotype (Mailing et al., 2019; Clauss et al., 2021; Huang et al., 2022). It has been shown that the gut lumen is physiologically maintained anaerobic. This acts as a selective pressure promoting the colonisation of anaerobic bacteria which ferment complex carbohydrates producing salubrious metabolites that the host can exploit. This is therefore conducive to a diverse and healthy gut microbiome repertoire (Wilde et al., 2024). However, many authors prescind from the critically important behaviour of exercise and its impact on gut oxygen tension. Exercise causes physiological gut hypoxia and thus potentiates the luminal anaerobic process, promoting anaerobic fermentative bacteria (Mailing et al., 2019; Clauss et al., 2021; Huang et al., 2022; Wilde et al., 2024). In a recent instructive review Wilde et al. conclude that “*the anerobic environment and the provision of complex carbohydrates favors microbes that ferment the carbohydrates into products that the host can use*”. Sedentary lifestyles and processed Western diets, nimious in oligosaccharides, undermine and compromise this process.

## A20

In addition to the host prioritising palatability over gut faunal health, the first act of a human host is to actually cede control to gut microbiota by means of inducing tolerance via A20 [tumour necrosis factor, alpha-induced protein 3 (TNFAIP_3_)]. Wang et al. showed that A20 was responsible for the tolerance of the gut to bacterial lipopolysaccharide (LPS) (Wang et al., 2009). Indeed, the enzyme was both necessary and sufficient for the process. A20-deficient mice showed marked bowel inflammation in response to LPS. The group also observed, in their murine model, that A20 levels were low at birth but rose, with increasing exposure to bacterial LPS, in the perinatal period. Interestingly, A20 was also low following bacterial eradication with antibiotics.

Gut flora have been incontrovertibly linked to obesity. This is either due to the composition of the gut microbiota, their effect on nutrient bio-availability and short-chain fatty acid production, or LPS-induced inflammation (Turnbaugh et al., 2009; DiBaise et al., 2012; Ridaura et al., 2013; Moreno-Indias et al., 2014). A20 enterocyte knockout mice exhibit a relative increase in *Firmicutes* gut bacteria and a relative fall in *Bacteriodetes* compared with wild-type mice (Vereecke et al., 2014). This is an enteric microbial profile seen in genetically obese (*ob)* mice (Ley et al., 2005). Inflammation in the gut and white adipose tissue has been strongly linked to obesity (Hand et al., 2015; Marchesi et al., 2015). It has also been implicated in the putative patho-mechanism of insulin resistance/diabetes spectrum endocrinopathy (Moreno-Indias et al., 2014). The microbiome in obesity is pro-inflammatory (DiBaise et al., 2012; Moreno-Indias et al., 2014; Scheithauer et al., 2020). The shift and narrowing in the gut microbiome repertoire during the process of “gut eutrophication” results in an immune response to the ascendancy of a new bacterial cohort. Furthermore, a diet replete with processed food, predominantly comprising unfixed nutrient mono- and oligomers, is thought to undermine the integrity of the alimentary canal barrier and increase gut permeability to LPS which elicits inflammatory cascades in white adipose tissue, germane to adipose tissue dysfunction, insulin resistance, and obesity (DiBaise et al., 2012; Moreno-Indias et al., 2014; Scheithauer et al., 2020). In addition, the expansion of pro-inflammatory bacteria is at the expense of anti-inflammatory microbiota such as *F. prausnitzii* which may putatively produce inflammatory repressors such as the short-chain fatty acid butyrate (Scheithauer et al., 2020).

A20, by dampening inflammation, has been shown to be necessary to maintain bowel wall integrity in inflammation (Mailing et al., 2019). Without it, LPS potentially gains systemic access through a permeable gut wall. It has been shown in a cohort of obese patients that there is an inverse correlation between the extent of A20 expression in adipose tissue and insulin resistance (Wang et al., 2009). A20 is also implicated by the fact that it appears to be part of a bacterial composition at birth and in early life that is related to obesity (DiBaise et al., 2012; Moreno-Indias et al., 2014). Hence, A20 potentially plays a role in tolerance, in early life, to bowel symbionts; defects may increase the risk of obesity.

## A20 and gut iatrogenic dysbiosis

Much of modern human existence and even medical intervention can tend to foment suboptimal faunal profiles. For example, much of the colonisation of the infantile gut and the respiratory tract occurs during vaginal delivery. Children who are born by a Caesarean section do not undergo the process to the same degree (Mueller et al., 2015a). It is believed that vaginal delivery activates A20 and a Caesarean section tends not to do so. One would hypothesise that a Caesarean section would be associated with obesity. This link is irrefutable (Li et al., 2013; Darmasseelane et al., 2014; Kuhle et al., 2015; Ardic et al., 2021; Chiavarini et al., 2023). A Caesarean section is also strongly linked to asthma (Huang et al., 2015; Suárez-Martínez et al., 2024). Consistent with this finding, children at risk of asthma have a paucity of *Lachnospira*, *Veillonella*, *Faecalibacterium*, and *Rothia* gut commensals, which may be a feature of Caesarean delivery (Arrieta et al., 2015). Furthermore, maternal faecal transplants in the form of a milkshake to their newborns delivered by a Caesarean section, result in more salubrious gut microbial profile in the infants (Lenharo, 2024).

Intriguingly, maternal antibiotics in the second and third trimester, but not the first, also increase the risk of childhood obesity (Mueller et al., 2015b). Asthma risk is also increased by maternal antibiotics (Marchesi et al., 2015; Suárez-Martínez et al., 2024; Tai et al., 2024). Differences in gut fauna have also been observed in children that are breastfed compared to those that are bottle fed (Yan et al., 2014). The latter technique is sterile while lactation necessarily involves greater exposure of the gut to bacteria. According to the A20 paradigm, breastfeeding would be associated with a reduction in obesity rates. This has been confirmed in meta-analyses (Yan et al., 2014).

## Collateral confirmation

Recent research on racehorses found that reduced diversity of the gut microbiome, early in life as foals, either due to antibiotics or as a feature of intraspecific variation, was associated with inferior athletic performance and increased risk of asthma in adulthood (Leng et al., 2024). Gracner et al. reported almost the obverse phenomenon in humans. Intriguingly, they observed that the omission or restriction of sugar from children for the first 1,000 days of life, including the time from conception to birth, significantly reduces the risk of metabolic disease in adulthood, including diabetes and hypertension (Gracner et al., 2024). It is *prima fascie* perplexing how intrauterine rationing could be eu-metabolic in adulthood. However, it accounted for a third of the risk reduction. This phenomenon is easily explicated by the gut eutrophication hypothesis. An aglycemic diet shapes the maternal gut microbiome,promoting diversity and is protective against eutrophic profiles. This is “inherited” by the child at birth as they egress the birth canal and come in contact with the maternal perineum and attendant fauna. In over 95% of births, the baby emerges from the birth canal in an occiput anterior position with the child’s face orientated posteriorly with respect to the mother (Gardberg and Tuppurainen, 1994). It is thought that in addition to facilitating foetal egress due to the human sacro-pelvic bauplan, it may equally promote parturient materno-fetal transplantation of gut microbiota (Mitteroecker and Fischer, 2024). As such, we anticipate that children born in a cephalic occiput anterior position will have a more salubrious gut microbiome profile and thus favourable metabolic profiles in adulthood than those born in a cephalic occiput posterior position with the face anteriorly orientated as the child is delivered.

In the context of a eutrophic diet, gut commensals prevent the propagation of pathogenic strains. Furuichi et al, using a murine model, report that a consortium of commensal bacteria impedes the proliferation of *Klebisella pneumonia* by resource restriction. However, this effect was found to be abrogated by an eutrophic diet by enriching the mice comestibles with glucose juxta-metabolites such as gluconate (Furuichi et al., 2024). This is a consistent finding (Pamer, 2024). An infusion of galactose precursors/juxta-metabolites eliminates the capacity of *Klebsiella michiganensis* to inhibit the growth of injurious *Escherichia coli* strains (Oliveira et al., 2020). An identical phenomenon was demonstrated with regard to the repression of *Salmonella* by resource sequestration by E. *coli* and *Klebsiella oxytoca* (Eberl et al., 2021; Osbelt et al., 2021).

In summary, a processed food diet and a paucity of exercise are important factors in gut eutrophication. This is characterised by overgrowth of certain strains that at least contribute to dysmetabolic states and a lack of faunal diversity. A reduction in the heterogeneity of the microbiome is seen in a number of dysbioses and has been linked to adverse health and performance outcomes.

## Data Availability

The original contributions presented in the study are included in the article/supplementary material. Further inquiries can be directed to the corresponding author.

## References

[B1] Agirman G. Hsiao E. Y. (2022). Gut microbes shape athletic motivation. Nature. 612, 633–634. doi: 10.1038/d41586-022-04355-3 36517676

[B2] Ardic C. Usta O. Omar E. Yıldız C. Memis E. (2021). Caesarean delivery increases the risk of overweight or obesity in 2-year-old children. J. Obstet Gynaecol. 41, 374–379. doi: 10.1080/01443615.2020.1803236 33063571

[B3] Arrieta M. C. Stiemsma L. T. Dimitriu P. A. Thorson L. Russell S. Yurist-Doutsch S.. (2015). Early infancy microbial and metabolic alterations affect risk of childhood asthma. Sci. Trans. Med. 7, 307ra152. doi: 10.1126/scitranslmed.aab2271 26424567

[B4] Chiavarini M. De Socio B. Giacchetta I. Fabiani R. (2023). Overweight and obesity in adult birth by cesarean section: A systematic review with meta-analysis. J. Public Health Manag Pract. 29, 128–141. doi: 10.1097/PHH.0000000000001687 36715592

[B5] Clauss M. Gérard P. Mosca A. Leclerc M. (2021). Interplay between exercise and gut microbiome in the context of human health and performance. Front. Nutr. 8, 637010. doi: 10.3389/fnut.2021.637010 34179053 PMC8222532

[B6] Darmasseelane K. Hyde M. J. Santhakumaran S. Gale C. Modi N. (2014). Mode of delivery and offspring body mass index, overweight and obesity in adult life: a systematic review and meta-analysis. PLoS One 9, e87896. doi: 10.1371/journal.pone.0087896 24586295 PMC3935836

[B7] DiBaise J. K. Frank D. N. Mathur R. (2012). Impact of the gut microbiota on the development of obesity: current concepts. Am. J. Gastroenterol. Supplements. 1, 22. doi: 10.1038/ajgsup.2012.5

[B8] Do M. H. Lee E. Oh M. J. Kim Y. Park H. Y. (2018). High-glucose or -fructose diet cause changes of the gut microbiota and metabolic disorders in mice without body weight change. Nutrients 10, 761. doi: 10.3390/nu10060761 29899272 PMC6024874

[B9] Dohnalová L. Lundgren P. Carty J. R. E. Goldstein N. Wenski S. L. Nanudorn P.. (2022). A microbiome-dependent gut-brain pathway regulates motivation for exercise. Nature. 612, 739–747. doi: 10.1038/s41586-022-05525-z 36517598 PMC11162758

[B10] Eberl C. Weiss A. S. Jochum L. M. Durai Raj A. C. Ring D. Hussain S.. (2021). coli enhance colonization resistance against Salmonella Typhimurium by competing for galactitol, a context-dependent limiting carbon source. Cell Host Microbe 29, 1680–1692.e7. doi: 10.1016/j.chom.2021.09.004 34610296

[B11] Furuichi M. Kawaguchi T. Pust M. M. Yasuma-Mitobe K. Plichta D. R. Hasegawa N.. (2024). Commensal consortia decolonize Enterobacteriaceae via ecological control. Nature 633, 878–886. doi: 10.1038/s41586-024-07960-6 39294375 PMC11424487

[B12] Gardberg M. Tuppurainen M. (1994). Einwirkung der persistierenden hinteren Hinterhauptslage auf den Geburtsverlauf [Effects of persistent occiput posterior presentation on mode of delivery. Z Geburtshilfe Perinatol. 198, 117–119. 7975796

[B13] Gracner T. Boone C. Gertler P. J. (2024). Exposure to sugar rationing in the first 1000 days of life protected against chronic disease. Science 31, eadn5421. doi: 10.1126/science.adn5421 PMC12238948 39480913

[B14] Hand L. E. Usan P. Cooper G. J. Xu L. Y. Ammori B. Cunningham P. S.. (2015). Adiponectin induces A20 expression in adipose tissue to confer metabolic benefit. Diabetes. 64, 128–136. doi: 10.2337/db13-1835 25190567 PMC4396702

[B15] Huang L. Chen Q. Zhao Y. Wang W. Fang F. Bao Y. (2015). Is elective cesarean section associated with a higher risk of asthma? A meta-analysis. J. Asthma. 1), 16–25. doi: 10.3109/02770903.2014.952435 25162303

[B16] Huang L. Li T. Zhou M. Deng M. Zhang L. Yi L.. (2022). Hypoxia improves endurance performance by enhancing short chain fatty acids production via gut microbiota remodeling. Front. Microbiol. 12, 820691. doi: 10.3389/fmicb.2021.820691 35197946 PMC8859164

[B17] Kawano Y. Edwards M. Huang Y. Bilate A. M. Araujo L. P. Tanoue T.. (2022). Microbiota imbalance induced by dietary sugar disrupts immune-mediated protection from metabolic syndrome. Cell. 185, 3501–3519. doi: 10.1016/j.cell.2022.08.005 36041436 PMC9556172

[B18] Kuhle S. Tong O. S. Woolcott C. G. (2015). Association between caesarean section and childhood obesity: a systematic review and meta-analysis. Obes. Rev. 16, 295–303. doi: 10.1111/obr.2015.16.issue-4 25752886

[B19] Leng J. Moller-Levet C. Mansergh R. I. O'Flaherty R. Cooke R. Sells P.. (2024). Early-life gut bacterial community structure predicts disease risk and athletic performance in horses bred for racing. Sci. Rep. 14, 17124. doi: 10.1038/s41598-024-64657-6 39112552 PMC11306797

[B20] Lenharo M. (2024). 'Poo milkshake' boosts the microbiome of c-section babies. Nature 635(8037):17–18. doi: 10.1038/d41586-024-03449-4 39448827

[B21] Ley R. E. Bäckhed F. Turnbaugh P. Lozupone C. A. Knight R. D. Gordon J. I. (2005). Obesity alters gut microbial ecology. Proc. Natl. Acad. Sci. 102, 11070–11075. doi: 10.1073/pnas.0504978102 16033867 PMC1176910

[B22] Li H. T. Zhou Y. B. Liu J. M. (2013). The impact of cesarean section on offspring overweight and obesity: a systematic review and meta-analysis. Int. J. Obes. 37, 893–899. doi: 10.1038/ijo.2012.195 23207407

[B23] Mailing L. J. Allen J. M. Buford T. W. Fields C. J. Woods J. A. (2019). Exercise and the gut microbiome: A review of the evidence, potential mechanisms, and implications for human health. Exerc Sport Sci. Rev. 47, 75–85. doi: 10.1249/JES.0000000000000183 30883471

[B24] Marchesi J. R. Adams D. H. Fava F. Hermes G. D. Hirschfield G. M. Hold G.. (2015). The gut microbiota and host health: a new clinical frontier. Gut. 65(2):330–9. doi: 10.1136/gutjnl-2015-309990 26338727 PMC4752653

[B25] Mitteroecker P. Fischer B. (2024). Evolution of the human birth canal. Am. J. Obstet Gynecol. 230, S841–S855. doi: 10.1016/j.ajog.2022.09.010 38462258

[B26] Moreno-Indias I. Cardona F. Tinahones F. J. Queipo-Ortuño M. I. (2014). Impact of the gut microbiota on the development of obesity and type 2 diabetes mellitus. Front. Microbiol. 5, 190. doi: 10.3389/fmicb.2014.00190 24808896 PMC4010744

[B27] Mueller N. T. Bakacs E. Combellick J. Grigoryan Z. Dominguez-Bello M. G. (2015a). The infant microbiome development: mom matters. Trends Mol. Med. 21, 109–117. doi: 10.1016/j.molmed.2014.12.002 25578246 PMC4464665

[B28] Mueller N. T. Whyatt R. Hoepner L. Oberfield S. Dominguez-Bello M. G. Widen E. M.. (2015b). Rundle A.Prenatal exposure to antibiotics, cesarean section and risk of childhood obesity. Int. J. Obes. (Lond) 39, 665–670. doi: 10.1038/ijo.2014.180 25298276 PMC4390478

[B29] Oliveira R. A. Ng K. M. Correia M. B. Cabral V. Shi H. Sonnenburg J. L.. (2020). Klebsiella michiganensis transmission enhances resistance to Enterobacteriaceae gut invasion by nutrition competition. Nat. Microbiol. 5, 630–641. doi: 10.1038/s41564-019-0658-4 31959968

[B30] Osbelt L. Wende M. Almási É Derksen E. Muthukumarasamy U. Lesker T. R.. (2021). Klebsiella oxytoca causes colonization resistance against multidrug-resistant K. pneumoniae in the gut via cooperative carbohydrate competition. Cell Host Microbe 29, 1663–1679.e7. doi: 10.1016/j.chom.2021.09.003 34610293

[B31] Pamer E. G. (2024). Gut microbes fend off harmful bacteria by depriving them of nutrients. Nature 633, 774–775. doi: 10.1038/d41586-024-02803-w 39294279

[B32] Ridaura V. K. Faith J. J. Rey F. E. Cheng J. Duncan A. E. Kau A. L.. (2013). Gut microbiota from twins discordant for obesity modulate metabolism in mice. Science. 341, 1241214. doi: 10.1126/science.1241214 24009397 PMC3829625

[B33] Ross F. C. Patangia D. Grimaud G. Lavelle A. Dempsey E. M. Ross R. P.. (2024). The interplay between diet and the gut microbiome: Implications for health and disease. Nat. Rev. Microbiol. 22(11), 671–86. doi: 10.1038/s41579-024-01068-4 39009882

[B34] Satokari R. (2020). High intake of sugar and the balance between pro- and anti-inflammatory gut bacteria. Nutrients. 12, 1348. doi: 10.3390/nu12051348 32397233 PMC7284805

[B35] Scheithauer T. P. M. Rampanelli E. Nieuwdorp M. Vallance B. A. Verchere C. B. van Raalte D. H.. (2020). Gut microbiota as a trigger for metabolic inflammation in obesity and type 2 diabetes. Front. Immunol. 11, 571731. doi: 10.3389/fimmu.2020.571731 33178196 PMC7596417

[B36] Suárez-Martínez C. Santaella-Pascual M. Yagüe-Guirao G. García-Marcos L. Ros G. Martínez-Graciá C. (2024). The early appearance of asthma and its relationship with gut microbiota: A narrative review. Microorganisms. 12, 1471. doi: 10.3390/microorganisms12071471 39065238 PMC11278858

[B37] Tai S. K. Lin Y. H. Lin C. H. Lin M. C. (2024). Antibiotic exposure during pregnancy increases risk for childhood atopic diseases: a nationwide cohort study. Eur. J. Med. Res. 29, 189. doi: 10.1186/s40001-024-01793-9 38504329 PMC10953187

[B38] Takeuchi T. Kubota T. Nakanishi Y. Tsugawa H. Suda W. Kwon A. T.. (2023). Gut microbial carbohydrate metabolism contributes to insulin resistance. Nature. 621, 389–395. doi: 10.1038/s41586-023-06466-x 37648852 PMC10499599

[B39] Turnbaugh P. J. Hamady M. Yatsunenko T. Cantarel B. L. Duncan A. Ley R. E.. (2009). A core gut microbiome in obese and lean twins. nature. 457, 480–484. doi: 10.1038/nature07540 19043404 PMC2677729

[B40] Vereecke L. Vieira-Silva S. Billiet T. van Es J. H. Mc Guire C. Slowicka K.. (2014). A20 controls intestinal homeostasis through cell-specific activities. Nat. Commun. 5, 5103. doi: 10.1038/ncomms6103 25267258

[B41] Wang J. Ouyang Y. Guner Y. Ford H. R. (2009). Grishin AV.Ubiquitin-editing enzyme A20 promotes tolerance to lipopolysaccharide in enterocytes. J. Immunol. 183, 1384–1392. doi: 10.4049/jimmunol.0803987 19570823 PMC2739219

[B42] Wilde J. Slack E. Foster K. R. (2024). Host control of the microbiome: Mechanisms, evolution, and disease. Science 385, eadi3338. doi: 10.1126/science.adi3338 39024451

[B43] Yan J. Liu L. Zhu Y. Huang G. (2014). Wang PP.The association between breastfeeding and childhood obesity: a meta-analysis. BMC Public Health 14, 1267. 25495402 10.1186/1471-2458-14-1267 PMC4301835

[B44] Zoetendal E. G. Raes J. Van Den Bogert B. Arumugam M. Booijink C. C. Troost F. J.. (2012). The human small intestinal microbiota is driven by rapid uptake and conversion of simple carbohydrates. ISME J. 6, 1415–1426. doi: 10.1038/ismej.2011.212 22258098 PMC3379644

